# *CCR5* Promoter Polymorphisms Associated With Pulmonary Tuberculosis in a Chinese Han Population

**DOI:** 10.3389/fimmu.2020.544548

**Published:** 2021-02-19

**Authors:** Shuyuan Liu, Nannan Liu, Hui Wang, Xinwen Zhang, Yufeng Yao, Shuqiong Zhang, Li Shi

**Affiliations:** ^1^ Institute of Medical Biology, Chinese Academy of Medical Sciences & Peking Union Medical College, Kunming, China; ^2^ The Third People’s Hospital of Kunming, Kunming, China; ^3^ Yunnan Key Laboratory of Vaccine Research and Development on Severe Infectious Diseases, Kunming, China

**Keywords:** tuberculosis, CCR5, polymorphism, susceptibility, Chinese Han

## Abstract

**Background:**

Tuberculosis (TB), an infectious disease caused by *Mycobacterium tuberculosis*, is a major public health concern. Chemokines and their receptors, such as RANTES, CXCR3, and CCR5, have been reported to play important roles in cell activation and migration in immune responses against TB infection.

**Methods:**

To understand the correlations involving *CCR5* gene variations, *M. tuberculosis* infection, and TB disease progression, a case-control study comprising 450 patients with TB and 306 healthy controls from a Chinese Han population was conducted, along with the detection of polymorphisms in the *CCR5* promoter using a sequencing method.

**Results:**

After adjustment for age and gender, the results of logistic analysis indicated that the frequency of rs2734648-G was significantly higher in the TB patient group (*P* = 0.002, OR = 1.38, 95% CI: 1.123–1.696); meanwhile, rs2734648-GG showed notable susceptibility to TB (*P* = 6.32E-06, OR = 2.173, 95% CI: 1.546–3.056 in a recessive model). The genotypic frequency of rs1799987 also varied between the TB and control groups (*P* = 0.008). In stratified analysis, rs2734648-GG significantly increased susceptibility to pulmonary TB in a recessive model (*P* < 0.0001, OR = 2.382, 95% CI: 1.663–3.413), and the rs2734648-G allele significantly increased susceptibility to TB recurrence in a dominant model (*P* = 0.0032, OR = 1.936, 95% CI: 1.221–3.068), whereas rs1799987-AA was associated with susceptibility to pulmonary TB (*P* = 0.0078, OR = 1.678, 95% CI: 1.141–2.495 in a recessive model) but not with extra-pulmonary TB and TB recurrence. A haplotype constructed with the major alleles of the eight SNPs in the *CCR5* promoter (rs2227010-rs2856758-rs2734648-rs1799987-rs1799988-rs41469351-rs1800023-rs1800024: A-A-G-G-T-C-G-C) exhibited extraordinarily increased risk of susceptibility to TB and pulmonary TB (*P* = 6.33E-11, OR = 24.887, 95% CI: 6.081–101.841).

**Conclusion:**

In conclusion, *CCR5* promoter polymorphisms were found to be associated with pulmonary TB and TB progression in Chinese Han people.

## Introduction

Tuberculosis (TB) is an infectious disease caused by *Mycobacterium tuberculosis* (*M. tuberculosis*). To date, TB remains a major public health concern since approximately one third of the world’s population has been infected with *M. tuberculosis* (1). Approximately 5% of the individuals who become infected with *M. tuberculosis* will develop clinical TB disease within 2 years of infection (2). TB is classified as primary TB or latent TB infection (2). Approximately 5–10% of the individuals with latent TB will develop clinical TB disease during their lifetime (2–4).

It was believed that active TB development was probably caused by the failure of host inflammatory responses against the pathogen (5). However, studies have identified complex interactions involving *M. tuberculosis* and the environment, along with the host genetic background playing a critical role in the outcome of *M. tuberculosis* exposure and TB development (4). A number of immunity-related genes have been reportedly associated with susceptibility to TB among different populations, such as human leukocyte antigen (HLA), low molecular weight polypeptide/transporter with antigen processing, natural resistance-associated macrophage protein 1 (NRAMP1), dendritic cell-specific ICAM-3-grabbing non-integrin (DC-SIGN), Toll-like receptors (TLR) 1 and 2, vitamin D receptor (VDR), TNF, interleukin (IL) -1β, IL-6, IL-8, IL-10, interferon γ (IFN-γ), and nucleotide oligomerization binding domain 2 (NOD2) (2, 4, 6, 7). Chemokines such as chemokine (C-C motif) ligand 2 (CCL-2)/monocyte chemoattractant protein 1 (MCP-1), RANTENS (*CCL5*), and chemokine C-X-C motif ligand 10 (CXCL10) have been found to be relevant in *M. tuberculosis* infection (7).

C-C motif chemokine receptor type 5 (CCR5), a transmembrane G-coupled cell-surface chemokine receptor, binds to different kinds of CC-chemokines, including human macrophage inflammatory protein-1α (MIP-1α), MIP-1β, RANTES (regulated on activation normal T cell expressed and secreted factor), monocyte chemotactic protein 1 (MCP-1), MCP-2, MCP-3, and MCP-4 (8). Previous studies report that *CCR5* is highly expressed during activation of T helper 1 (Th1) cells, which play a critical role in host immune responses against TB (9). Moreover, *CCR5* expression is found to be substantially increased during *M. tuberculosis* infection (10–12). Pokkali et al. found that *CCR5* expression is significantly higher in pulmonary TB patients compared to that in healthy controls (10), and Qiu et al. reported that *CCR5* expression levels in rhesus monkeys with severe TB disease exhibit remarkably up-regulated lymphocytes in the lungs, bronchial lymph nodes, and spleen (11). Additionally, CCR5 and its ligand play important roles in T-cell activation and migration during immune responses against TB infection. Galkina et al. observed that CCR5^-/-^ CD8^+^ T cells exhibit an approximately 50% reduction in effector CD8^+^ T cell transmigration from pulmonary vascular compartments into interstitial compartments, as compared with CCR5 wild-type CD8^+^ T cells (13). CCR5 has also been found to possibly regulate effector CD8^+^ T cell contraction and memory generation after *M. tuberculosis* infection (14). Furthermore, several studies have suggested that CXCR3, CCR5, and CXCR6 potentially mediate *M. tuberculosis*-specific CD4^+^T cell migration out of the vascular endothelium, and their entry into the lungs during *M. tuberculosis* infection, which is a critical step in host immune responses against *M. tuberculosis* infection (15). Therefore, CCR5 seems to play an important role in the immune response against *M. tuberculosis* infection.

Although CCR5 is reported to be involved in resistance against *M. tuberculosis* infection, a number of studies have reported the association of *CCR5* gene variants with TB infection and progression. In 2014, Carpenter et al. (16) performed an analysis regarding the possible associations between rs1799987 of *CCR5* and clinically active TB phenotypes in three different populations (Peru, Xhosa, and Colored), but found no significant associations. In this study, we examined genetic polymorphisms in the *CCR5* promoter in the Chinese Han population to investigate the association between *CCR5* promoter polymorphisms and *M. tuberculosis* infection, and TB progression.

## Methods and Materials

### Subjects

A total of 450 patients with TB, including 325 cases of pulmonary TB (PTB) and 125 cases of extrapulmonary TB (EPTB, which was defined as TB influencing extrapulmonary sites such as lymph nodes, abdomen, urinary tract, skin, joints, bones, and meninges, exclusively or in combination with PTB), who were enrolled in the Third People’s Hospital of Kunming between 2018–2019, were selected as a TB patient group for this study. All subjects were genetically unrelated and belonged to the Chinese Han population from Yunnan province (southwest China).

Diagnoses of TB were based on clinical case definition guidelines for TB issued by the World Health Organization (WHO) (17), Diagnosis for Pulmonary Tuberculosis (WS 288-2017) (18) and Classification of Tuberculosis (WS 196-2017) (19) from the Health Industry Standard of the People’s Republic of China. The diagnostic criteria were as follows: (1) *M. tuberculosis* positively confirmed by sputum smear culture bacteriological assessment; (2) clinical symptoms such as cough, fever and weight loss over two weeks, and chest X-ray consistent with TB disease. Usually, Tuberculin skin test (TST) and interferon-γ release assay (IGRA) are also positive. Patients with immunodeficiency, autoimmune diseases, or other acute or chronic infections were excluded from this study.

Over the same period, 306 healthy individuals were recruited as a control group. All the controls had negative history for TB disease and were without any acute or chronic pulmonary disorder, or any bacterial or viral infection or other immune-mediated disorders. All the controls were self-reported Han Chinese.

### DNA Extraction and Sequencing

Two to 5 ml of peripheral blood was drawn from each participant, and genomic DNA was extracted from peripheral lymphocytes using the QIAamp Blood Mini Kit (Qiagen, Hilden, Germany) in a Biosafety Level 2 Laboratory of the Third People’s Hospital of Kunming. The *CCR5* promoter region was PCR amplified using primers used in a previously published study (20); CCR5P_F: 5’-gacgagaaagctgagggtaaga-3’ and CCR5P_R: 5’-taaccgtctgaaactcattcca-3’. The amplified PCR fragment was 1388 bp in length. PCR for each sample was carried out using the TAKARA PrimeSTAR Max DNA polymerase kit (TAKARA, Dalian, China) in 50 µl reaction volumes containing 10 ng genomic DNA, 10 pmol of each primer, 25 µl 2 × PrimeSTAR Max Premix (TAKARA). Amplification consisted of an initial denaturation step of 5 min at 98°C, followed by: 30 cycles of denaturation for 10 s at 98°C, 5 s of annealing at 55°C, extension at 72°C for 90 s, and a final extension for 5 min at 72°C. Purified PCR fragments were subjected to Sanger DNA sequencing to detect the sites of polymorphism using the Big Dye Terminator Reaction Mix (Applied Biosystems Foster City, CA, USA), along with the same primers used for PCR amplification. Sequencing reaction products were purified using the Big Dye Terminator Purification Kit (Applied Biosystems) and run on an ABI 3730XL sequencer. Sequencing data were analyzed using the DNASTAR Lasergene v.7.1 package. All the *CCR5* promoter region sequencing data in this study have been deposited in the Figshare database named “*CCR5* promoter sequences of TB patients and controls” (DOI: 10.6084/m9.figshare.12015624).

### Polymorphic Loci in CCR5 Promoter

In our previous study (20), we found that there are nine identified single nucleotide polymorphisms (SNPs) loci located in the *CCR5* gene promoter. Six SNPs, rs2227010 (A>G), rs2734648 (T>G), rs1799987 (G>A), rs1799988 (T>G), rs1800023 (G>A), and rs1800024 (C>T) were found to be polymorphic in the Chinese Han population sample, whereas the three remaining sites, rs2856758 (AA), rs41469351 (CC), and rs41355345 (CC), were monomorphic in this sample. Thus, we analyzed association between alleles and genotypes of these six SNPs with TB.

### Statistical Analysis

The distribution of age and sex between the case and control groups was compared *via* Student’s *t*-test and χ^2^-tests in SPSS (v.19.0; SPSS Inc., Chicago, IL, USA). Basic statistical analyses for allelic and genotypic frequencies of the six SNPs were carried out using PLINK v.1.9 (http://zzz.bwh.harvard.edu/plink/data.shtml) (21), and risks were estimated using odds ratios (OR) with 95% confidence intervals (95% CI). A goodness-of-fit χ^2^-test was used to test for Hardy-Weinberg equilibrium (HWE) for each SNP in the control group, with a threshold of 0.05, which was also assessed using PLINK.

The Linkage disequilibrium (LD) and haplotype frequencies (deduced from the phenotype) among eight SNPs (rs2227010-rs2856758-rs2734648-rs1799987-rs1799988-rs41469351-rs1800023-rs1800024) were calculated based on the genotype results using a standard Expectation Maximization (ignoring missing data) algorithm with a partition-ligation approach for blocks by Haploview v.4.2 software (22). Haploview calculates the LD coefficient D’, LOD and r^2^ between each pair of genetic markers. *D’* values were defined in the range [-1, 1], with a value of 1 representing perfect disequilibrium. A *D’* value over 0.8 indicated there is a strong linkage disequilibrium among SNPs. The lowest frequency threshold for haplotype analysis was 0.01, and hhaplotype with frequency less than this number will not be considered in analysis. The differences in the haplotypes (with frequencies over 0.01) between the TB and control groups, between PTB and control groups, as well as between EPTB and control groups were determined by χ^2^-test. And risks were estimated using ORs and 95%CI. OR and 95% CI were used to estimate associations between SNPs and TB disease by adjusting for age and gender using multivariate logistic regression models. The threshold for statistical significance was *P <*0.05. Bonferroni correction was applied for multiple comparisons among alleles and genotypes, and the *P*-value was adjusted to 0.05/n. Power-analysis was performed using Power and Sample Size Calculations (v.3.1.2) (23).

## Results

### Characteristics of Subjects

**Table 1** presents participant demographic characteristic data, including gender, age, and clinical type of TB. The mean age of the TB group was 43.76 ± 16.01 years, with the sex ratio (male/female) being 249/201, while the mean age of the control group was 44.68 ± 9.26 years, with a sex ratio (male/female) of 154/152. The distributions of age and gender between the TB and control groups showed no statistical differences (*P* > 0.05). The mean ages were 45.31 ± 15.82 years in the PTB group and 39.75 ± 15.86 years in the EPTB group; whereas the sex ratio (male/female) was 190/135 in the PTB group and 59/66 in the EPTB group. For the initial treatment (IT) and retreatment (RT) groups, the mean ages were 43.20 ± 16.35 years and 44.68 ± 15.46 years, respectively, and sex ratios (male/female) were 144/135 in the IT and 105/66 in the RT group (*P* = 0.043).

**Table 1:** Demographic and clinical data for tuberculosis patients and controls.

Study groups		Num.	Sex, M:F	Age (mean ± SD), years
**TB**		450	249:201	43.76 ± 16.01
	**PTB**	325	190:135	45.31 ± 15.82
	Infiltration	146	80:66	45.97 ± 16.92
	Secondary	161	106:55	44.50 ± 14.80
	Cavity	18	4:14	47.17 ± 15.99
	**EPTB**	125	59: 66	39.75 ± 15.86
	Lymph nodes	13	7:6	36.31 ± 13.68
	Genitourinary tract	31	7:24	40.48 ± 14.66
	bone and joint	24	13:11	41.58 ± 15.20
	cutaneous	5	1:4	42.20 ± 21.81
	celiac	14	8:6	41.00 ± 23.29
	Meningitis	8	8:0	34.13 ± 10.87
	Peritoneal	2	1:1	20.50 ± 6.36
	Pleuritis	1	0:1	47.00
	Multi-site concurrency	17	8:9	39.53 ± 13.01
	others	10	6:4	42.60 ± 18.91
	Initial treatment	279	144:135	43.20 ± 16.35
	Retreatment	171	105:66	44.68 ± 15.46
**Healthy control**		306	154:152	44.68 ± 9.26

Num., number; M, male; F, female; SD, standard deviation; TB, tuberculosis; PTB, pulmonary tuberculosis; EPTB, extrapulmonary tuberculosis.

### Comparisons of Allelic and Genotypic Frequencies of CCR5 Promoter SNPs Between TB Patients and Controls

All six *CCR5* promoter SNPs with polymorphism exhibited HWE in the control group (*P* > 0.05). However, in the TB patient group, rs2734648, rs1799987, and rs1800023 were not in HWE. The allelic and genotypic frequencies of the six *CCR5* promoter SNPs were compared between the TB patient and control groups, after adjusting for age and gender based on the logistic regression model (**Table 2**). The results showed that the frequency of rs2734648-G was significantly higher in the TB patient group compared to that in the control group (*P* = 0.002, OR = 1.380, 95% CI: 1.123–1.696); the genotypic distribution of rs2734648 was significantly different between the TB and control groups (*P* = 1.07E-05). Furthermore, we performed inheritance model analysis and found that rs2734648-GG was significantly associated with TB disease, and exhibited 2-fold susceptibility in a recessive inheritance model (*P* = 6.32E-06, OR = 2.173, 95% CI: 1.546–3.056). The genotypic distribution of rs1799987 also showed significant difference between the TB and control groups (*P* = 0.008).

**Table 2:** Comparison of allelic and genotypic frequencies of CCR5 gene between TB patients and controls.

Comparison		Control (n = 306)	TB (n = 450)	*P*	OR (95%CI)
		n (Freq.)	n (Freq.)		
**rs2227010**					
**Allelic**	**A**	501 (0.819)	734(0.816)		Ref.
	**G**	111 (0.181)	166(0.184)	0.879	1.021(0.783–1.332)
**Genotypic**	**A/A**	207 (0.676)	302(0.671)	0.988	Ref.
	**G/A**	87 (0.284)	130(0.289)		1.024(0.741–1.416)
	**G/G**	12 (0.039)	18(0.040)		1.028(0.485–2.180)
**Dominant**	**A/A**	207 (0.676)	302(0.671)		Ref.
	**G/G-G/A**	99 (0.324)	148 (0.329)	0.877	1.025(0.752–1.397)
**Recessive**	**A/A-G/A**	294 (0.961)	432 (0.960)		Ref.
	**G/G**	12 (0.039)	18(0.040)	0.957	1.021(0.484–2.151)
**rs2734648**					
**Allelic**	**T**	328 (0.536)	410(0.456)		Ref.
	**G**	284 (0.464)	490(0.544)	**0.002**	1.380(1.123–1.696)
**Genotypic**	**T/T**	83 (0.271)	118(0.262)	**1.07E-05**	Ref.
	**G/T**	162 (0.529)	174(0.387)		0.755(0.531–1.075)
	**G/G**	61 (0.199)	158(0.351)		1.822(1.212–2.739)
**Dominant**	**T/T**	83 (0.271)	118(0.262)		Ref.
	**G/G-G/T**	223 (0.729)	332 (0.738)	0.783	1.048(0.754–1.454)
**Recessive**	**G/T-T/T**	245 (0.801)	292 (0.649)		Ref.
	**G/G**	61 (0.199)	158(0.351)	**6.32E-06**	2.173(1.546–3.056)
**rs1799987**					
**Allelic**	**G**	365 (0.596)	516 (0.573)		Ref.
	**A**	247 (0.404)	384 (0.427)	0.372	1.100 (0.893–1.355)
**Genotypic**	**G/G**	110 (0.359)	174 (0.387)	**0.008**	Ref.
	**G/A**	145 (0.474)	168 (0.373)		0.732 (0.529–1.015)
	**A/A**	51 (0.167)	108 (0.240)		1.339 (0.889–2.017)
**Dominant**	**G/G**	110 (0.359)	174 (0.387)		Ref.
	**A/A-A/G**	196 (0.641)	276 (0.613)	0.449	0.890 (0.659–1.203)
**Recessive**	**G/G-A/G**	255 (0.833)	342 (0.760)		Ref.
	**A/A**	51 (0.167)	108(0.240)	0.015	1.579 (1.090–2.287)
**rs1799988**					
**Allelic**	**T**	358 (0.585)	532 (0.591)		Ref.
	**C**	254 (0.415)	368 (0.409)	0.8117	0.975 (0.791–1.201)
**Genotypic**	**T/T**	107 (0.350)	157 (0.349)	0.8806	Ref.
	**C/T**	144 (0.471)	218 (0.484)		1.032 (0.747–1.426)
	**C/C**	55 (0.180)	75 (0.167)		0.929 (0.607–1.423)
**Dominant**	**T/T**	107 (0.350)	157 (0.349)		Ref.
	**C/C-C/T**	199 (0.650)	293 (0.651)	0.982	1.003 (0.740–1.361)
**Recessive**	**T/T-C/T**	251 (0.820)	375 (0.833)		Ref.
	**C/C**	55 (0.180)	75 (0.167)	0.6401	0.913 (0.622–1.339)
**rs1800023**					
**Allelic**	**G**	336 (0.549)	482 (0.536)		Ref.
	**A**	276 (0.451)	418 (0.464)	0.6061	1.056 (0.859–1.297)
**Genotypic**	**G/G**	88 (0.288)	119 (0.264)	0.7798	Ref.
	**A/G**	160 (0.523)	244 (0.542)		1.128 (0.803–1.585)
	**A/A**	58 (0.190)	87 (0.193)		1.109 (0.720–1.708)
**Dominant**	**G/G**	88 (0.288)	119 (0.264)		Ref.
	**A/A-A/G**	218 (0.712)	331 (0.736)	0.4838	1.123 (0.812–1.553)
**Recessive**	**A/G-G/G**	248 (0.810)	363 (0.807)		Ref.
	**A/A**	58 (0.190)	87 (0.193)	0.8966	1.025 (0.708–1.483)
**rs1800024**					
**Allelic**	**C**	462 (0.755)	687 (0.763)		Ref.
	**T**	150 (0.245)	213 (0.237)	0.7064	0.955 (0.751–1.214)
**Genotypic**	**C/C**	170 (0.556)	268 (0.596)	0.1254	Ref.
	**T/C**	122 (0.399)	151 (0.336)		1.789 (0.911–3.513)
	**T/T**	14 (0.046)	31 (0.069)		1.405 (0.726–2.717)
**Dominant**	**C/C**	170 (0.556)	268 (0.596)		Ref.
	**T/T-T/C**	136 (0.444)	182 (0.404)	0.2742	0.849 (0.633–1.139)
**Recessive**	**T/C-C/C**	292 (0.954)	419 (0.931)		Ref.
	**T/T**	14 (0.046)	31 (0.069)	0.1869	1.543 (0.807–2.952)

Co, control; TB, tuberculosis; OR, odds ratio; CI, confidence interval.

The P-value, OR, and 95% CIs of pair-wise comparisons between TB and control groups were calculated based on the logistic regression model adjusted for age and gender. Bonferroni correction was applied, and the P-value was adjusted to 0.008 (0.05/6). And the P-value lower than 0.008 were marked in bold.

This study had powers over 80% to detect ORs of 1.021 for rs2227010, 0.725 for rs2734648, and 0.975 for rs1799988, and had power of 53.8% to detect with an OR of 1.1 for rs1799987, 60.6% to detect with an OR of 1.056 for rs1800023, and 72.6% to detect with a OR of 0.955 for rs1800024, respectively, in 450 TB patients compared with 306 controls.

### Stratification Analysis of the Association Between TB and *CCR5* Promoter Polymorphisms

We performed stratification analysis to investigate the association of TB susceptibility with *CCR5* promoter SNPs. We stratified the TB patients into PTB and EPTB patients and compared the distribution of allelic and genotypic frequencies of the six SNPs among the stratification groups and the control group. The associations between SNPs and PTB or EPTB groups were adjusted for age and gender using multivariate logistic regression models. **Table 3** shows comparative results of rs2734648 and rs1799987. The results showed that the frequency of rs2734648-G was significantly higher in PTB patients as compared to controls (*P* = 0.0013, OR = 1.488, 95% CI: 1.192–1.858). Carriers of rs2734648-GG showed a notable increase in susceptibility to PTB in the recessive inheritance model (*P* < 0.0001, OR = 2.382, 95% CI: 1.663–3.413). Carriers of rs1799987-AA also showed a significant association with PTB in the recessive inheritance model (*P* = 0.0078, OR = 1.687, 95% CI: 1.141–2.495). However, significant associations of these two SNPs with EPTB in the recessive inheritance model were not detected (**Table 3**). For other SNPs, no significant differences were found between the PTB and control groups. It should be noted that the results from PTB (**Table 3**) simply reinforce the TB associations observed from **Table 2**, which is not surprising, since PTB represents 72.2% of the TB sample. Finally, no significant difference was found between the EPTB and control groups, and between the EPTB and PTB groups (**Supplementary Table 1**).

**Table 3:** Stratification analysis on the association between CCR5 promoter SNPs and clinical TB phenotypes.

Comparison		Control (n = 306)	PTB (n = 325)	EPTB (n = 125)	PTB vs Co.		EPTB vs Co.		EPTB vs PTB	
		n (Freq.)	n (Freq.)	n (Freq.)	*P*	OR (95%CI)	*P*	OR (95%CI)	*P*	OR (95%CI)
**rs2734648**										
**Allelic**	**T**	328(0.536)	284(0.437)	126(0.504)		Ref.		Ref.		Ref.
	**G**	284(0.464)	366(0.563)	124(0.496)	**1.3E-03**	1.488(1.192–1.858)	0.394	1.137(0.847–1.526)	0.700	0.764(0.570–1.023)
**Genotypic**	**T/T**	83(0.271)	80(0.246)	38 (0.304)	**5.20E-06**	Ref.	0.076	Ref.	0.256	Ref.
	**G/T**	162(0.530)	124(0.382)	50 (0.400)		0.794(0.540–1.168)		0.674(0.410–1.109)		0.849(0.511–1.409)
	**G/G**	61(0.199)	121(0.372)	37 (0.296)		2.058(1.332–3.179)		1.325(0.756–2.321)		0.644(0.378–1.098)
**Dominant**	**T/T**	83(0.271)	80(0.246)	38 (0.304)		Ref.		Ref.		Ref.
	**G/G-G/T**	223(0.729)	245(0.754)	87 (0.696)	0.5	1.140(0.798–1.628)	0.730	0.852(0.540–1.346)	0.211	0.748(0.473–1.181)
**Recessive**	**G/T-T/T**	245(0.801)	204(0.628)	88 (0.704)		Ref.		Ref.		Ref.
	**G/G**	61(0.199)	121(0.372)	37 (0.296)	**1.64E-06**	2.382(1.663–3.413)	0.049	1.689(1.050–2.717)	0.129	0.709(0.454–1.106)
**rs1799987**										
**Allelic**	**G**	365(0.596)	371(0.571)	145(0.580)		Ref.		Ref.		Ref.
	**A**	247(0.404)	279(0.429)	105(0.420)	0.519	1.111(0.888–1.390)	0.657	1.070(0.794–1.442)	0.802	0.963(0.717–1.294)
**Genotypic**	**G/G**	110(0.359)	128(0.394)	46 (0.368)	**0.0028**	Ref.	0.740	Ref.	0.353	Ref.
	**G/A**	145(0.474)	115(0.354)	53 (0.424)		0.682(0.479–0.970)		0.874(0.548–1.393)		1.282(0.803–2.049)
	**A/A**	51(0.167)	82(0.252)	26 (0.208)		1.382(0.897–2.130)		1.219(0.680–2.187)		0.882(0.506–1.537)
**Dominant**	**G/G**	110(0.359)	128(0.394)	46 (0.368)		Ref.		Ref.		Ref.
	**A/A-A/G**	196(0.641)	197(0.606)	79 (0.632)	0.373	0.864(0.626–1.193)	0.990	0.964(0.626–1.485)	0.614	1.116(0.729–1.709)
**Recessive**	**G/G-A/G**	255(0.833)	243(0.748)	99 (0.792)		Ref.		Ref.		Ref.
	**A/A**	51(0.167)	82(0.252)	26 (0.208)	**0.0078**	1.687(1.141–2.495)	0.470	1.313(0.776–2.223)	0.324	0.778(0.472–1.282)

Co, control; PTB, pulmonary tuberculosis; EPTB, extra pulmonary tuberculosis; OR, odds ratio; CI, confidence interval.

The P-value, OR, and 95% CIs of pair-wise comparisons between PTB and controls, and EPTB and controls, were calculated based on the logistic regression model adjusted for age and gender. Bonferroni correction was applied, and the P-value was adjusted to 0.008 (0.05/6). And the P-value lower than 0.008 were marked in bold.

Additionally, we stratified the TB patients into IT and RT subgroups according to disease stage at the time of treatment, and analyzed allelic and genotypic distributions of the six SNPs. Associations between SNPs and disease recurrence were adjusted for age and gender using multivariate logistic regression models. We found rs2734648-G and rs2734648-GG to be significantly associated with TB recurrence (**Table 4**). After comparison between IT and control, RT and control, and RT and IT groups, we found that rs2734648-GG was significantly associated with TB recurrence in a dominant inheritance model (*P* = 0.0032, OR = 1.936, 95% CI: 1.221–3.068), while rs1799987 showed no significant association with disease recurrence (**Table 4** and **Supplementary Table 2**).

**Table 4:** Association between CCR5 gene variants and TB recurrence.

Comparison		Co (n = 306)	IT (n = 279)	RT (n = 171)	*P*	IT vs Co	*P*	RT vs Co		*P*	IT vs RT
		n (freq.)	n (freq.)	n (freq.)		OR (95% CI)	**	OR (95% CI)		**	OR (95% CI)
**rs2734648**											
**Allelic**	T	328(0.536)	269(0.482)	141(0.412)		Ref.		Ref.			Ref.
	G	284(0.464)	289(0.518)	201(0.588)	0.0725	1.241(0.986–1.561)	**1.7E-04**	1.646(1.260–2.151)		0.0471	1.327(1.011–1.741)
**Genotypic**	T/T	83(0.271)	86(0.308)	32(0.187)	**1.0E-05**	Ref.	**2.0E-04**	Ref.		0.01	Ref.
	G/T	162(0.529)	97(0.348)	77(0.450)		0.578(0.390–0.856)		1.233(0.755–2.012)			2.133(1.289–3.532)
	G/G	61(0.199)	96(0.344)	62(0.363)		1.519(0.978–2.359)		2.636(1.537–4.522)			1.736(1.036–2.909)
**Dominant**	T/T	83(0.271)	86(0.308)	32(0.187)		Ref.		Ref.			Ref.
	G/G-G/T	223 (0.729)	193 (0.692)	139 (0.813)	0.35	0.835(0.584–1.195)	0.034	1.617(1.021–2.560)		**0.0032**	1.936(1.221–3.068)
**Recessive**	T/T-G/T	245 (0.801)	183 (0.656)	109(0.637)		Ref.		Ref.			Ref.
	G/G	61(0.199)	96(0.344)	62(0.363)	**1.00E-04**	2.107(1.450–3.062)	**1.00E-04**	2.285(1.502–3.475)		0.58	1.084(0.728–1.614)
**rs1799987**											
**Allelic**	G	365(0.596)	322(0.577)	194(0.567)		Ref.		Ref.			Ref.
	A	247(0.404)	236(0.423)	148(0.433)	0.555	1.803(0.858–1.367)	0.3848	1.127(0.862–1.474)	0.6775		1.041(0.793–1.366)
**Genotypic**	G/G	110(0.359)	115(0.412)	59(0.345)	**9.0E-04**	Ref.	0.51	Ref.	0.045		Ref.
	A/G	145(0.474)	92(0.330)	76(0.444)		0.607(0.419–0.878)		0.977(0.642–1.488)			1.610(1.040–2.492)
	A/A	51(0.167)	72(0.258)	36(0.211)		1.350(0.866–2.105)		1.316(0.774–1.577)			0.975(0.586–1.621)
**Dominant**	G/G	110(0.359)	115(0.412)	59(0.345)		Ref.		Ref.			Ref.
	A/A-A/G	196 (0.641)	164 (0.588)	112 (0.655)	0.19	0.800(0.573–1.117)	0.73	1.065(0.720–1.577)	0.11		1.331(0.897–1.976)
**Recessive**	A/G-G/G	255 (0.833)	207 (0.742)	135 (0.789)		Ref.		Ref.			Ref.
	A/A	51(0.167)	72(0.258)	36(0.211)	**7.9E-03**	1.739(1.162–2.602)	0.24	1.333(0.829–2.144)	0.3		0.767(0.486–1.208)

Co, control; IT, initial treatment; RT, retreatment; OR, odds ratio; CI, confidence interval.

The P-value, OR, and 95% CIs of pair-wise comparisons between IT and control, RT and control, and RT and IT groups were calculated on the basis of the logistic regression model adjusted for age and gender. Bonferroni correction was applied, and the P-value was adjusted to 0.008 (0.05/6). And the P-value lower than 0.008 were marked in bold.

### *CCR5* Promoter SNP Combination Analysis and Association With TB

LD among eight SNPs (rs2227010, rs2856758, rs2734648, rs1799987, rs1799988, rs41469351, rs1800023, rs1800024) in the *CCR5* promoter was estimated, where the LD coefficient D (*D’*) was calculated. The *D’* value of these eight SNPs was >0.8, indicating that these *CCR5* promoter SNPs were in LD (**Supplementary Figure 1**). Next, we constructed haplotypes of the eight SNPs (rs2227010-rs2856758-rs2734648-rs1799987-rs1799988-rs41469351-rs1800023-rs1800024) and compared haplotypes with frequencies over 0.01 between case and control groups, as listed in **Table 5**. The results revealed that haplotype H1 (A-A-T-G-T-C-G-C) was the most prevalent, both in control (50.3%) as well as TB patient groups (41.1%), and showed a significant resistance to TB disease (*P* = 1.17E-04, OR = 0.660, 95% CI: 0.535–0.816), with notable resistance to PTB (*P* = 1.05E-05, OR = 0.599, 95% CI: 0.477–0.753). In contrast, haplotype H5 (A-A-G-G-T-C-G-C) presented an extremely increased risk of susceptibility to TB by over 20-fold (*P* = 7.84E-10, OR = 21.877, 95% CI: 5.378–88.996), as well as a notably increased risk of susceptibility to PTB and EPTB (*P* = 6.33E-11, OR = 24.887, 95% CI: 6.081–101.841 and *P* = 8.35E-06, OR = 14.038, 95% CI: 3.088–63.804, respectively); whereas haplotype H7 (A-A-G-A-T-C-G-C) increased the risk of susceptibility to TB and PTB by 10-fold (*P* = 0.004, OR = 10.715, 95% CI: 1.421–80.796, and *P* = 0.002, OR = 12.196, 95% CI: 1.595–93.269, respectively). Additionally, both haplotypes H10 (A-A-T-A-T-C-G-C) and H11 (G-A-G-G-C-C-A-C) appeared only in the case group, mainly in the PTB cohort, and frequency differences between PTB and control groups were remarkable after Bonferroni correction (*P* = 7.63E-05 and *P* = 0.003, respectively). However, there was no difference between PTB and EPTB groups (**Supplementary Table 3**).

**Table 5:** *CCR5* promoter haplotype frequencies in case and control groups.

Haplotype^a,d^		Haplotype similarity^b^	Co (2n = 612)	TB patients (2n = 900)			PTB (2n = 650)			EPTB patients (2n = 250)		
			N (freq.)	N (freq.)	*P* ^c^	OR (95%CI)	N (freq.)	*P* ^c^	OR (95%CI)	N (freq.)	*P* ^c^	OR (95%CI)
H1:	A A T G T C G C	HHC	307.60(0.503)	370.30(0.411)	**1.17E-04**	0.660 (0.535–0.816)	250.99(0.386)	**1.05E-05**	0.599 (0.477–0.753)	121.5(0.486)	0.6652	0.947(0.706–1.271)
H2:	A A G A C C A T	HHF*1	126.14(0.206)	179.78(0.200)	0.644	0.941 (0.728–1.217)	128.64(0.198)	0.649	0.938 (0.711–1.237)	47.6(0.190)	0.5996	0.917(0.633–1.328)
H3:	G A G A C C A C	HHE	97.75(0.160)	143.12(0.159)	0.863	0.975 (0.736–1.293)	95.95(0.148)	0.499	0.899 (0.661–1.223)	45.9(0.184)	0.3999	1.183(0.804–1.740)
H4:	A A G G T C A C	HHA	29.97(0.049)	46.68(0.052)	0.857	1.044 (0.652–1.673)	38.87(0.060)	0.421	1.223 (0.749–1.997)	9.8(0.039)	0.5409	0.728(0.340–1.555)
H5:	A A G G T C G C	unknown	2.03(0.003)	61.78(0.069)	**7.84E-10**	21.877 (5.378–88.996)	49.95(0.077)	**6.33E-11**	24.887 (6.081–101.841)	11.2(0.045)	**8.35E-06**	14.038(3.088–63.804)
H6:	A A G G C C A T	unknown	1.95(0.003)	14.47(0.016)	0.019	5.030 (1.124–22.509)	13.46(0.021)	0.005	6.551 (1.453–29.528)	1(0.004)	0.8396	1.225(0.111–13.570)
H7:	A A G A T C G C	unknown	1.00(0.002)	15.81(0.018)	**4.10E-03**	10.715 (1.421–80.796)	12.88(0.020)	**0.002**	12.196 (1.595–93.269)	3(0.012)	0.0423	7.421(0.768–71.690)
H8:	A A G A C C G T	unknown	7.04(0.012)	4.36(0.005)	0.134	0.411 (0.124–1.365)	3.30(0.005)	0.200	0.435 (0.117–1.614)	1.6(0.006)	0.4821	0.697(0.144–3.379)
H9:	A A T G C C G C	unknown	7.07(0.012)	1.01(0.001)	0.006	0.094 (0.012–0.762)	1.01(0.002)	0.025	0.131 (0.016–1.063)	0(0.000)	0.46	0.302(0.038–2.426)
H10:	A A T A T C G C	unknown	0.00(0.000)	20.04(0.022)	**2.43E-04**	14.612(1.960–108.915)	17.24(0.027)	**7.63E-05**	17.459(2.324–131.181)	3(0.012)	0.027	9.746(1.095–86.770)
H11:	G A G G C C A C	unknown	0.00(0.000)	10.48(0.012)	0.034	7.576(0.976–58.835)	10.51(0.016)	**0.003**	11.530(1.495–88.938)	0(0.000)	–	–

^a^Haplotypes were constructed with rs2227010-rs2856758-rs2734648-rs1799987-rs1799988-rs41469351-rs1800023-rs1800024.

^b^HHA, HHB, HHC, and HHF were previously reported (25–27).

^c^Bonferroni correction was applied, and the P-value was adjusted to 0.004 (0.05/11). And the P-value lower than 0.004 were marked in bold.

^d^Haplotypes with frequencies over 0.01 were compared and listed in this table.

## Discussion

TB is a serious infectious disease caused by *M. tuberculosis*; however, only 5–10% of infected individuals actually develop the active form of disease with clinical symptoms, prompting researchers to identify the factors influencing susceptibility to TB. Understanding host immune responses to *M. tuberculosis* infection is critical in identifying the reasons behind varying outcomes after *M. tuberculosis* exposure (latent or active TB disease), and for the development of effective TB vaccines and immune therapeutics. There is substantial evidence to suggest that the onset of TB is influenced by host genetic factors (2, 9, 15, 24). CCR5 has been reported to play important roles in immune responses against *M. tuberculosis* infection by regulating and activating the recruitment of macrophages, and by further activation of T-cells. In the present study, we investigated associations between *CCR5* promoter polymorphisms and TB and discovered that *CCR5* promoter polymorphisms were significantly associated with PTB and TB progression in the Chinese Han population for the first time.

In this study, we found that the allelic frequency of rs2734648-G was significantly higher in the TB patient group, especially in the PTB group, as compared to the control group, and also that rs2734648-GG carriers had a 2.382-fold increased risk of susceptibility to PTB in a recessive inheritance model. Additionally, rs2734648 was found to be significantly associated with TB recurrence. It has been reported that *CCR5* variants may alter the response of CCR5-chemokines, including altered ligand-binding properties (28), and *CCR5* promoter polymorphisms could differentially affect *CCR5* gene transcription. We constructed a predictive model involving potential binding sites of transcription factors in the *CCR5* promoter and discovered that SNPs in the *CCR5* promoter might differentially influence transcription factor binding, based on the nucleotide substitution(s) involved (20). Mummidi et al. demonstrated that G to T substitution in rs2734648 (-2554G>T) is associated with differences in binding avidity of the NF-κB family of transcription factors (the binding avidity of rs2734648-G is greater than that of rs2734648-T), which might affect the transcriptional activity of *CCR5*. rs2856758-G can bind to novel nuclear factor 1 (NF1), which can repress transcription of certain genes (29), whereas rs2856758-A cannot bind to NF1 (26). For another SNP in the *CCR5* promoter, rs1799987-AA, we found an increasing risk of susceptibility to PTB in a recessive inheritance model. McDremott et al. reported that rs1799987 (-2459A>G) influences the expression of *CCR5*, and rs1799987-G has 45% lower promoter activity than rs1799987-A *in vitro* (30). The latter authors also observed that rs1799987-A/rs1799988-C in combination stimulates *CCR5* promoter activity by 45% more than other rs1799987/rs1799988 allelic combinations (30). Li et al. showed that rs1799988 C to T substitution results in reduced expression of *CCR5*, which consequently correlated with slower AIDS progression. Furthermore, rs1799988-CC carriers display increased CCR5 expression on the surface of peripheral blood mononuclear cells (PBMCs), CD4+ cells, and CD4+ monocytes, as compared to two other *CCR5*-rs1799988 genotypes (31). Therefore, we deduced that SNPs in the *CCR5* promoter could influence *CCR5* gene and cell surface CCR5 protein expression by altering the binding of transcription factors, thereby affecting the function of CCR5. Hence, rs2734648-GG and rs1799987-AA were significantly associated with PTB and TB progression by possibly increased expression of CCR5.

We also analyzed the effects of combinations of the 8 *CCR5* promoter SNPs (rs2227010, rs2856758, rs2734648, rs1799987, rs1799988, rs41469351, rs1800023, rs1800024) on TB susceptibility and found that haplotype H1 (A-A-T-G-T-C-G-C)—constructed using the major alleles of the eight SNPs—was significantly associated with resistance to PTB; and haplotype H5 (A-A-G-G-T-C-G-C) increased the susceptibility to PTB by over 20-times. *CCR2-CCR5* haplogroups constructed using CCR2(V>64I), rs2856758(-2733A>G), rs2734648(-2554G>T), rs1799987(-2459G>A), rs1799988(-2135T>C), rs41469351(-2132C>T), rs1800023(-2086A>G), rs1800024(-1835C>T), and rs333(CCR5Δ32) have been characterized and described in earlier studies, and the haplogroups are termed HHA, HHB, HHC, HHD, HHE, HHF*1, HHF*2, HHG*1, and HHG*2, respectively (25, 26). CCR2-CCR5 haplogroups are correlated with differences in *CCR5* expression and transcriptional activity. HHA is associated with lower *CCR5* expression, whereas HHF and HHG are associated with higher *CCR5* expression (32). Similarly, K562 cells, HHA and HHC exhibit lower transcriptional activity, whereas in Jurkat T-cells, HHB, and HHD show higher transcriptional activity than HHA (33, 34). In 2011, Mamtani et al. found that the *CCR5* promoter haplogroup HHD was associated with susceptibility to TB, by increasing *CCR5* expression in either activated PBMCs, or surface expression on activated (HLA-DR^+^) CD4^+^ T cells (34). In our study, we constructed haplotypes with eight SNPs in the *CCR5* promoter, and only rs2227010 was not included in the defined CCR2-CCR5 haplogroups. Our results showed that H1 was similar to HHC (which exhibited lower transcriptional activity and lower *CCR5* expression), H2 was similar to HHF*1 (related to higher *CCR5* expression), H3 was similar to HHE, and H4 was similar to HHA. Therefore, the noticeable protection against PTB by the H1 haplotype in this study was likely due to the lower transcriptional activity of H1 (similar to HHC, which was associated with lower CCR5 expression). However, haplotype H5 was different from haplotype H1 at only one locus: rs2734648, which was T in haplotype H1 and G in H5. Note that H5 is a novel haplotype not previously detected in any other population. Based on the significant susceptibility of rs2734648-G to TB, and the extraordinarily high frequency of rs2734648-G in the Han population, rs2734648-G as well as haplotype H5 could provide crucial insights into immune responses to TB in the Chinese Han population. In this study, we also found one haplotype rs2856758A-rs2734648T-rs1799987G-rs1799988T-rs41469531C-rs1800023A-rs1800024C was most similar as HHD. However, the haplotype frequencies were very low (0.5 and 0.5% in control and TB group respectively, and data not showed) and with no difference between TB and control group. In addition, haplotype H1, which presented only two differences loci when compared with HHD (rs41469351-C and rs1800023-G in H1, whereas rs41469351-T and rs1800023-A in HHD), showed significant frequency difference between TB and control groups (P = 2.25E-04, OR = 0.674, 95% CI: 0.547–0.832). But haplotype HHD and H1 showed entirely opposite effects on the susceptibility to TB. The reason of this discrepancy could be the different frequency of rs41469351 and rs1800023. According to the 1000 Genomes database the rs41469351-T is only detected in African and American population with the frequencies of 26 and 2%, respectively, and were monomorphic (rs41469351-CC) in Chinese Han and European people (http://asia.ensembl.org/Homo_sapiens/Variation/Population?db=core;r=3:46370271-46371271;v=rs41469351;vdb=variation;vf=5860779). And for rs1800023, the A-allele frequencies in Chinese Han people is 43%, however, there are 91% in African, 63% in European (http://asia.ensembl.org/Homo_sapiens/Variation/Population?db=core;r=3:46370317-46371317;v=rs1800023;vdb=variation;vf=816197). So rs41469351 and rs1800023 maybe important loci which might affect the susceptibility of TB. Haplotypes H7, H10, and H11, which were unlike any reported haplogroups, also showed significantly higher frequencies in the TB group, as well as the PTB cohort. Hence, the combined functions of *CCR5* promoter SNPs might play important roles in *CCR5*-mediated immune responses to TB.

Associations of SNPs with diseases have always been inconsistent among different populations. Previous studies indicate that *CCR5* allelic frequencies are remarkably different among different populations (16, 35), which might influence the results of correlative studies. Among most populations in the world (including Africans, Americans, Europeans, Japanese, and South Asians), rs2734648-G is the major allele; however, in Chinese Han populations, rs2734648-T is the predominant allele (Han Chinese in Beijing, and Southern Han Chinese from the 1000 Genomes database, and Chinese Han in Yunnan in this study), indicating that the frequency differences in rs2734648 could account for the specific association with TB in Han Chinese people. However, the function of rs2734648 substitution is still unclear; therefore, functional studies regarding the role of rs2734648 in *M. tuberculosis* infection and TB progression need to be conducted in the future.

Hence, more studies involving more individuals from different populations are needed. Additionally, as discussed above, SNP combinations also play an important role in susceptibility to TB and in its progression. Hence, further studies regarding haplotype structure, especially in terms of combinations of rs2734648 with other SNPs in the *CCR5* promoter, are required.

## Conclusions

SNP rs2734638-G of the *CCR5* promoter, as well as haplotype H5, consistent with rs2734648-G, are significantly associated with susceptibility to PTB and with TB recurrence by affecting the transcriptional activity and expression of *CCR5*.

## Data Availability Statement

The data sets presented in this study can be found in online repositories. The names of the repository/repositories and accession number(s) can be found below: https://figshare.com/, 10.6084/m9.figshare.12015624.

## Ethics Statement

The studies involving human participants were reviewed and approved by the institutional review board of the Third Hospital of Kunming (approval number is 2018030720). The patients/participants provided their written informed consent to participate in this study.

## Author Contributions

SL and LS conceived and designed the research. SL and NL mainly performed experiments and data analysis. HW and SZ did the clinical diagnosis. HW and XZ prepared samples for experiments and performed part of the experiments. SL wrote the manuscript. YY, SZ, and LS wrote parts of the manuscript and reviewed the manuscript. All authors contributed to the article and approved the submitted version.

## Funding

This work was supported by the National Natural and Science Foundation of China (31401063); and the Special Funds for high-level health talents of Yunnan Province (D-201669, L-201615, and H-2018014). The funders had no role in the design of the study, data collection and analysis, decision to publish, or preparation of the manuscript.

## Conflict of Interest

The authors declare that the research was conducted in the absence of any commercial or financial relationships that could be construed as a potential conflict of interest.
