# Supplementary material for: CCR5 Promoter Polymorphisms Associated With Pulmonary Tuberculosis in a Chinese Han Population
Source: Front Immunol. 2021 Feb 19;11:544548. doi: 10.3389/fimmu.2020.544548 (PMC7935552; doi:10.3389/fimmu.2020.544548)
Supplement: Supplementary file 1 [file Table_1.docx]

**Supplementary** **Material**

**sTable 1 Stratification analysis on the association between CCR5 promoter SNPs and clinical phenotypes of TB**

| **Comparison** | | **Co(n=306)** | **PTB(n=325)** | **EPTB(n=125)** |  | **PTB vs Co** | |  | **EPTB vs Co** | |  | **EPTB vs PTB** | |
| --- | --- | --- | --- | --- | --- | --- | --- | --- | --- | --- | --- | --- | --- |
|  |  | **n(Freq.)** | **n(Freq.)** | **n(Freq.)** |  | ***P*** | **OR (95%CI)** |  | ***P*** | **OR (95%CI)** |  | ***P*** | **OR (95%CI)** |
| **rs2227010** | | | | | | | | | | | | | |
| **Allelic** | **A** | 501(0.819) | 532(0.818) | 202(0.808) |  |  | Ref. |  |  | Ref. |  |  | Ref. |
|  | **G** | 111(0.181) | 118(0.182) | 48(0.192) |  | 0.677 | 1.083(0.746-1.572) |  | 0.715 | 1.073(0.736-1.562) |  | 0.717 | 1.071(0.738-1.555) |
| **Genotypic** | **A/A** | 207(0.676) | 218 (0.671) | 84 (0.672) |  | 0.905 | Ref. |  | 0.850 | Ref. |  | 0.528 | Ref. |
|  | **G/A** | 87(0.285) | 96 (0.295) | 34 (0.272) |  |  | 1.048(0.741-1.482) |  |  | 0.963(0.602-1.542) |  |  | 0.919(0.577-1.464) |
|  | **G/G** | 12(0.039) | 11 (0.034) | 7 (0.056) |  |  | 0.870(0.376-2.016) |  |  | 1.438(0.547-3.777) |  |  | 1.652(0.620-4.403) |
| **Dominant** | **A/A** | 207(0.676) | 218 (0.671) | 84 (0.672) |  |  | Ref. |  |  | Ref. |  |  | Ref. |
|  | **G/G-G/A** | 99(0.324) | 107 (0.329) | 41 (0.328) |  | 0.879 | 1.026(0.736-1.432) |  | 0.880 | 1.021(0.665-1.590) |  | 0.980 | 0.994(0.641-1.543) |
| **Recessive** | **A/A-G/A** | 294(0.961) | 314 (0.966) | 118 (0.944) |  |  | Ref. |  |  | Ref. |  |  | Ref. |
|  | **G/G** | 12(0.039) | 11 (0.034) | 7 (0.056) |  | 0.719 | 0.858(0.373-1.975) |  | 0.570 | 1.453(0.559-3.782) |  | 0.283 | 1.693(0.641-4.471) |
| **rs2734648** | | | | | | | | | | | | | |
| **Allelic** | **T** | 328(0.536) | 284(0.437) | 126(0.504) |  |  | Ref. |  |  | Ref. |  |  | Ref. |
|  | **G** | 284(0.464) | 366(0.563) | 124(0.496) |  | 0.0013 | 1.488(1.192-1.858) |  | 0.394 | 1.137(0.847-1.526) |  | 0.700 | 0.764(0.570-1.023) |
| **Genotypic** | **T/T** | 83(0.271) | 80(0.246) | 38 (0.304) |  | 5.20E-06 | Ref. |  | 0.076 | Ref. |  | 0.256 | Ref. |
|  | **G/T** | 162(0.530) | 124(0.382) | 50 (0.400) |  |  | 0.794(0.540-1.168) |  |  | 0.674(0.410-1.109) |  |  | 0.849(0.511-1.409) |
|  | **G/G** | 61(0.199) | 121(0.372) | 37 (0.296) |  |  | 2.058(1.332-3.179) |  |  | 1.325(0.756-2.321) |  |  | 0.644(0.378-1.098) |
| **Dominant** | **T/T** | 83(0.271) | 80(0.246) | 38 (0.304) |  |  | Ref. |  |  | Ref. |  |  | Ref. |
|  | **G/G-G/T** | 223(0.729) | 245(0.754) | 87 (0.696) |  | 0.5 | 1.140(0.798-1.628) |  | 0.730 | 0.852(0.540-1.346) |  | 0.211 | 0.748(0.473-1.181) |
| **Recessive** | **G/T-T/T** | 245(0.801) | 204(0.628) | 88 (0.704) |  |  | Ref. |  |  | Ref. |  |  | Ref. |
|  | **G/G** | 61(0.199) | 121(0.372) | 37 (0.296) |  | 1.64E-06 | 2.382(1.663-3.413) |  | 0.049 | 1.689(1.050-2.717) |  | 0.129 | 0.709(0.454-1.106) |
| **rs1799987** | | | | | | | | | | | | | |
| **Allelic** | **G** | 365(0.596) | 371(0.571) | 145(0.580) |  |  | Ref. |  |  | Ref. |  |  | Ref. |
|  | **A** | 247(0.404) | 279(0.429) | 105(0.420) |  | 0.519 | 1.111(0.888-1.390) |  | 0.657 | 1.070(0.794-1.442) |  | 0.802 | 0.963(0.717-1.294) |
| **Genotypic** | **G/G** | 110(0.359) | 128(0.394) | 46 (0.368) |  | 0.0028 | Ref. |  | 0.740 | Ref. |  | 0.353 | Ref. |
|  | **G/A** | 145(0.474) | 115(0.354) | 53 (0.424) |  |  | 0.682(0.479-0.970) |  |  | 0.874(0.548-1.393) |  |  | 1.282(0.803-2.049) |
|  | **A/A** | 51(0.167) | 82(0.252) | 26 (0.208) |  |  | 1.382(0.897-2.130) |  |  | 1.219(0.680-2.187) |  |  | 0.882(0.506-1.537) |
| **Dominant** | **G/G** | 110(0.359) | 128(0.394) | 46 (0.368) |  |  | Ref. |  |  | Ref. |  |  | Ref. |
|  | **A/A-A/G** | 196(0.641) | 197(0.606) | 79 (0.632) |  | 0.373 | 0.864(0.626-1.193) |  | 0.990 | 0.964(0.626-1.485) |  | 0.614 | 1.116(0.729-1.709) |
| **Recessive** | **G/G-A/G** | 255(0.833) | 243(0.748) | 99 (0.792) |  |  | Ref. |  |  | Ref. |  |  | Ref. |
|  | **A/A** | 51(0.167) | 82(0.252) | 26 (0.208) |  | 0.0078 | 1.687(1.141-2.495) |  | 0.470 | 1.313(0.776-2.223) |  | 0.324 | 0.778(0.472-1.282) |
| **rs1799988** | | | | | | | | | | | | | |
| **Allelic** | **T** | 358(0.585) | 379(0.583) | 153(0.612) |  |  | Ref. |  |  | Ref. |  |  | Ref. |
|  | **C** | 254(0.415) | 271(0.417) | 97(0.388) |  | 0.946 | 1.008(0.806-1.261) |  | 0.464 | 0.894(0.661-1.207) |  | 0.429 | 0.887(0.658-1.195) |
| **Genotypic** | **T/T** | 107(0.350) | 110 (0.339) | 47 (0.376) |  | 0.895 | Ref. |  | 0.770 | Ref. |  | 0.725 | Ref. |
|  | **C/T** | 144(0.470) | 159 (0.489) | 59 (0.472) |  |  | 1.074(0.758-1.522) |  |  | 0.933(0.590-1.474) |  |  | 0.868(0.552-1.367) |
|  | **C/C** | 55(0.180) | 56 (0.172) | 19 (0.152) |  |  | 0.990(0.627-1.565) |  |  | 0.786(0.421-1.468) |  |  | 0.794(0.426-1.480) |
| **Dominant** | **T/T** | 107(0.350) | 110 (0.339) | 47 (0.376) |  |  | Ref. |  |  | Ref. |  |  | Ref. |
|  | **C/C-C/T** | 199(0.650) | 215 (0.661) | 78 (0.624) |  | 0.767 | 1.051(0.757-1.460) |  | 0.780 | 0.892(0.580-1.374) |  | 0.454 | 0.849(0.553-1.304) |
| **Recessive** | **T/T-C/T** | 251(0.820) | 269 (0.828) | 106 (0.848) |  |  | Ref. |  |  | Ref. |  |  | Ref. |
|  | **C/C** | 55(0.180) | 56 (0.172) | 19 (0.152) |  | 0.806 | 0.950(0.631-1.431) |  | 0.488 | 0.818(0.463-1.445) |  | 0.034 | 1.821(1.040-3.189) |
| **rs1800023** | | | | | | | | | | | | | |
| **Allelic** | **G** | 336(0.549) | 340(0.523) | 142(0.568) |  |  | Ref. |  |  | Ref. |  |  | Ref. |
|  | **A** | 276(0.451) | 310(0.477) | 108(0.432) |  | 0.356 | 1.110(0.889-1.385) |  | 0.611 | 0.926(0.688-1.246) |  | 0.226 | 0.834(0.622-1.119) |
| **Genotypic** | **G/G** | 88(0.288) | 79 (0.243) | 40 (0.320) |  | 0.444 | Ref. |  | 0.930 | Ref. |  | 0.249 | Ref. |
|  | **A/G** | 160(0.522) | 182 (0.560) | 62 (0.496) |  |  | 1.267(0.875-1.836) |  |  | 0.853(0.530-1.371) |  |  | 0.673(0.417-1.084) |
|  | **A/A** | 58(0.190) | 64 (0.197) | 23 (0.184) |  |  | 1.229(0.770-1.962) |  |  | 0.872(0.474-1.607) |  |  | 0.710(0.386-1.306) |
| **Dominant** | **G/G** | 88(0.288) | 79 (0.243) | 40 (0.320) |  |  | Ref. |  |  | Ref. |  |  | Ref. |
|  | **A/A-A/G** | 218(0.712) | 246 (0.757) | 85 (0.680) |  | 0.205 | 1.257(0.882-1.791) |  | 0.710 | 0.858(0.547-1.345) |  | 0.097 | 0.682(0.434-1.074) |
| **Recessive** | **A/G-G/G** | 248(0.810) | 261 (0.803) | 102 (0.816) |  |  | Ref. |  |  | Ref. |  |  | Ref. |
|  | **A/A** | 58(0.190) | 64 (0.197) | 23 (0.184) |  | 0.815 | 1.048(0.706-1.557) |  | 0.810 | 0.946(0.565-1.647) |  | 0.756 | 0.920(0.542-1.560) |
| **rs1800024** | | | | | | | | | | | | | |
| **Allelic** | **C** | 462(0.755) | 490(0.754) | 197(0.788) |  |  |  |  |  | Ref. |  |  | Ref. |
|  | **T** | 150(0.245) | 160(0.246) | 53(0.212) |  | 0.965 | 1.006(0.778-1.300) |  | 0.299 | 0.829(0.581-1.182) |  | 0.280 | 0.824(0.580-1.171) |
| **Genotypic** | **C/C** | 170(0.556) | 192 (0.591) | 76 (0.608) |  | 0.049 |  |  | 0.620 | Ref. |  | 0.152 | Ref. |
|  | **T/C** | 122(0.398) | 106 (0.326) | 45 (0.360) |  |  | 0.769(0.552-1.072) |  |  | 0.825(0.534-1.276) |  |  | 1.072(0.692-1.663) |
|  | **T/T** | 14(0.046) | 27 (0.083) | 4 (0.032) |  |  | 1.708(0.867-3.363) |  |  | 0.639(0.204-2.006) |  |  | 0.374(0.127-1.106) |
| **Dominant** | **C/C** | 170(0.556) | 192 (0.591) | 76 (0.608) |  |  |  |  |  | Ref. |  |  | Ref. |
|  | **T/T-T/C** | 136(0.444) | 133 (0.409) | 49 (0.392) |  | 0.371 | 0.866(0.631-1.187) |  | 0.400 | 0.806(0.527-1.232) |  | 0.739 | 0.931(0.610-1.419) |
| **Recessive** | **T/C-C/C** | 292(0.954) | 298 (0.917) | 121 (0.968) |  |  |  |  |  | Ref. |  |  | Ref. |
|  | **T/T** | 14(0.046) | 27 (0.083) | 4 (0.032) |  | 0.057 | 1.890(0.971-3.676) |  | 0.517 | 0.689(0.222-2.137) |  | 0.055 | 0.365(0.125-1.065) |

**Abbreviations:** Co, control; Freq., frequency; PTB, pulmonary tuberculosis; EPTB, extra pulmonary tuberculosis; OR, odds ratio; CI, confidence interval.

Note: The *P*-value, OR and 95%CIs of pairs comparison between PTB and control and EPTB and control were calculated based on the logistic regression model adjusted by age and gender. Bonferroni correction was applied and the *P*-value was adjusted to 0.008 (0.05/6). And the *P*-value lower than 0.008 were marked in bold.

**sTable 2 The Association between CCR5 gene variants and TB recurrence**

| **Comparison** | | **Co (n=306)** | **IT (n=279)** | **RT(n=171)** |  | **IT vs Co** | |  | **RT vs Co** | |  | **IT vs RT** | |
| --- | --- | --- | --- | --- | --- | --- | --- | --- | --- | --- | --- | --- | --- |
|  |  | **n(Freq.)** | **n(freq.)** | **n(freq.)** |  | **P-value** | **OR (95% CI)** |  | **P-value** | **OR (95% CI)** |  | **P-value** | **OR (95% CI)** |
| **rs2227010** | | | | | | | | | | | | | |
| **Allelic** | **A** | 501(0.819) | 465(0.833) | 269(0.787) |  |  | Ref. |  |  | Ref. |  |  | Ref. |
|  | **G** | 111(0.181) | 93(0.177) | 73(0.213) |  | 0.508 | 0.903(0.667-1.222) |  | 0.228 | 1.225(0.880-1.704) |  | 0.079 | 1.357(0.965-1.909) |
| **Genotypic** | **A/A** | 207(0.676) | 194 (0.695) | 108 (0.632) |  | 0.71 | Ref. |  | 0.44 | Ref. |  | 0.13 | Ref. |
|  | **G/A** | 87(0.285) | 77 (0.276) | 53 (0.310) |  |  | 0.944(0.656-1.359) |  |  | 1.168(0.772-1.765) |  |  | 1.236(0.811-1.885) |
|  | **G/G** | 12(0.039) | 8 (0.029) | 10 (0.058) |  |  | 0.711(0.285-1.777) |  |  | 1.597(0.669-3.816) |  |  | 2.245(0.861-5.859) |
| **Dominant** | **A/A** | 207(0.676) | 194 (0.695) | 108 (0.632) |  |  | Ref. |  |  | Ref. |  |  | Ref. |
|  | **G/G-G/A** | 99(0.324) | 85 (0.305) | 63 (0.368) |  | 0.623 | 0.916(0.646-1.300) |  | 0.321 | 1.220(0.824-1.806) |  | 0.162 | 1.331(0.891-1.990) |
| **Recessive** | **A/A-G/A** | 294(0.961) | 271 (0.971) | 161 (0.942) |  |  | Ref. |  |  | Ref. |  |  | Ref. |
|  | **G/G** | 12(0.039) | 8 (0.029) | 10 (0.058) |  | 0.483 | 0.723(0.291-1.796) |  | 0.336 | 1.522(0.643-3.600) |  | 0.117 | 2.104(0.814-5.440) |
| **rs2734648** | | | | | | | | | | | | | |
| **Allelic** | **T** | 328(0.536) | 269(0.482) | 141(0.412) |  |  | Ref. |  |  | Ref. |  |  | Ref. |
|  | **G** | 284(0.464) | 289(0.518) | 201(0.588) |  | 0.066 | 1.241(0.986-1.561) |  | **1.7E-04** | 1.646(1.260-2.151) |  | 0.047 | 1.327(1.011-1.741) |
| **Genotypic** | **T/T** | 83(0.271) | 86 (0.308) | 32 (0.187) |  | **1.0E-05** | Ref. |  | **2.0E-04** | Ref. |  | 0.01 | Ref. |
|  | **G/T** | 162(0.530) | 97 (0.348) | 77 (0.450) |  |  | **0.578(0.390-0.856)** |  |  | 1.233(0.755-2.012) |  |  | 2.133(1.289-3.532) |
|  | **G/G** | 61(0.199) | 96 (0.344) | 62 (0.363) |  |  | 1.519(0.978-2.359) |  |  | 2.636(1.537-4.522) |  |  | 1.736(1.036-2.909) |
| **Dominant** | **T/T** | 83(0.271) | 86 (0.308) | 32 (0.187) |  |  | Ref. |  |  | Ref. |  |  | Ref. |
|  | **G/G-G/T** | 223(0.729) | 193 (0.692) | 139 (0.813) |  | 0.324 | 0.835(0.584-1.195) |  | 0.034 | 1.617(1.021-2.560) |  | **0.0032** | **1.936(1.221-3.068)** |
| **Recessive** | **G/T-T/T** | 245(0.801) | 183 (0.656) | 109 (0.637) |  |  | Ref. |  |  | Ref. |  |  | Ref. |
|  | **G/G** | 61(0.199) | 96 (0.344) | 62 (0.363) |  | **1.00E-04** | **2.017(1.450-3.062)** |  | **1.00E-04** | **2.285(1.502-3.475)** |  | 0.58 | 1.084(0.728-1.614) |
| **rs1799987** | | | | | | | | | | | | | |
| **Allelic** | **G** | 365(0.596) | 322(0.577) | 194(0.567) |  |  | Ref. |  |  | Ref. |  |  | Ref. |
|  | **A** | 247(0.404) | 236(0.423) | 148(0.433) |  | 0.555 | 1.083(0.858-1.367) |  | 0.3848 | 1.127(0.862-1.474) |  | 0.678 | 1.041(0.793-1.366) |
| **Genotypic** | **G/G** | 110(0.359) | 115 (0.412) | 59 (34.5%) |  | **9.0E-04** | Ref. |  | 0.51 | Ref. |  | 0.045 | Ref. |
|  | **G/A** | 145(0.474) | 92 (0.330) | 76 (44.4%) |  |  | 0.607(0.419-0.878) |  |  | 0.977(0.642-1.488) |  |  | 1.610(1.040-2.492) |
|  | **A/A** | 51(0.167) | 72 (0.258) | 36 (21.1%) |  |  | 1.350(0.866-2.105) |  |  | 1.316(0.774-1.577) |  |  | 0.975(0.586-1.621) |
| **Dominant** | **G/G** | 110(0.359) | 115 (0.412) | 59 (34.5%) |  |  | Ref. |  |  | Ref. |  |  | Ref. |
|  | **A/A-A/G** | 196(0.641) | 164 (0.588) | 112 (65.5%) |  | 0.191 | 0.800(0.573-1.117) |  | 0.73 | 1.065(0.720-1.577) |  | 0.11 | 1.331(0.897-1.976) |
| **Recessive** | **G/G-A/G** | 255(0.833) | 207 (0.742) | 135 (79%) |  |  | Ref. |  |  | Ref. |  |  | Ref. |
|  | **A/A** | 51(0.167) | 72 (0.258) | 36 (21.1%) |  | **7.9E-03** | **1.739(1.162-2.602)** |  | 0.24 | 1.333(0.829-2.144) |  | 0.3 | 0.767(0.486-1.208) |
| **rs1799988** | | | | | | | | | | | | | |
| **Allelic** | **T** | 358(0.585) | 335(0.600) | 197(0.576) |  |  | Ref. |  |  | Ref. |  |  | Ref. |
|  | **C** | 254(0.415) | 223(0.400) | 145(0.424) |  | 0.593 | 0.938(0.743-1.185) |  | 0.788 | 1.037(0.793-1.356) |  | 0.471 | 1.106(0.841-1.453) |
| **Genotypic** | **T/T** | 107(0.350) | 105 (0.376) | 52 (0.304) |  | 0.8 | Ref. |  | 0.26 | Ref. |  | 0.087 | Ref. |
|  | **C/T** | 144(0.470) | 125 (0.448) | 93 (0.544) |  |  | 0.885(0.617-1.268) |  |  | 1.329(0.872-2.026) |  |  | 1.502(0.980-2.303) |
|  | **C/C** | 55(0.180) | 49 (0.176) | 26 (0.152) |  |  | 0.908(0.568-1.452) |  |  | 0.973(0.549-1.724) |  |  | 1.071(0.600-1.914) |
| **Dominant** | **T/T** | 107(0.350) | 105 (0.376) | 52 (0.304) |  |  | Ref. |  |  | Ref. |  |  | Ref. |
|  | **C/C-C/T** | 199(0.650) | 174 (0.624) | 119 (0.696) |  | 0.503 | 0.891(0.636-1.249) |  | 0.311 | 1.230(0.823-1.839) |  | 0.069 | 1.381(0.920-2.073) |
| **Recessive** | **T/T-C/T** | 251(0.820) | 230 (0.824) | 145 (0.848) |  |  | Ref. |  |  | Ref. |  |  | Ref. |
|  | **C/C** | 55(0.180) | 49 (0.176) | 26 (0.152) |  | 0.897 | 0.972(0.636-1.487) |  | 0.44 | 0.818(0.492-1.362) |  | 0.515 | 0.842(0.501-1.414) |
| **rs1800023** | | | | | | | | | | | | | |
| **Allelic** | **G** | 336(0.549) | 307(0.550) | 175(0.512) |  |  | Ref. |  |  | Ref. |  |  | Ref. |
|  | **A** | 276(0.451) | 251(0.450) | 167(0.488) |  | 0.968 | 0.995(0.790-1.253 |  | 0.268 | 1.162(0.891-1.514) |  | 0.261 | 1.167(0.891-1.529) |
| **Genotypic** | **G/G** | 88(0.288) | 84 (0.301) | 35 (0.205) |  | 0.83 | Ref. |  | 0.096 | Ref. |  | 0.03 | Ref. |
|  | **A/G** | 160(0.522) | 139 (0.498) | 105 (0.614) |  |  | 0.910(0.625-1.325) |  |  | 1.650(1.039-2.621) |  |  | 1.813(1.135-2.897) |
|  | **A/A** | 58(0.190) | 56 (0.201) | 31 (0.181) |  |  | 1.011(0.630-1.624) |  |  | 1.344(0.748-2.415) |  |  | 1.329(0.737-2.397) |
| **Dominant** | **G/G** | 88(0.288) | 84 (0.301) | 35 (0.205) |  |  | Ref. |  |  | Ref. |  |  | Ref. |
|  | **A/A-A/G** | 218(0.712) | 195 (0.699) | 136 (0.795) |  | 0.721 | 0.937(0.656-1.338) |  | 0.042 | 1.569(1.004-2.451) |  | 0.019 | 1.674(1.066-2.628) |
| **Recessive** | **A/G-G/G** | 248(0.810) | 223 (0.799) | 140 (0.819) |  |  | Ref. |  |  | Ref. |  |  | Ref. |
|  | **A/A** | 58(0.190) | 56 (0.201) | 31 (0.181) |  | 0.733 | 1.074(0.713-1.617) |  | 0.824 | 0.947(0.584-1.534) |  | 0.612 | 0.882(0.542-1.435) |
| **rs1800024** | | | | | | | | | | | | | |
| **Allelic** | **C** | 462(0.755) | 422(0.756) | 265(0.775) |  |  | Ref. |  |  | Ref. |  |  | Ref. |
|  | **T** | 150(0.245) | 136(0.244) | 77(0.225) |  | 0.957 | 0.993(0.760-1.296) |  | 0.488 | 0.895(0.654-1.225) |  | 0.524 | 0.902(0.655-1.240) |
| **Genotypic** | **C/C** | 170(0.556) | 162 (0.581) | 106 (62%) |  | 0.28 | Ref. |  | 0.18 | Ref. |  | 0.8 | Ref. |
|  | **T/C** | 122(0.398) | 98 (0.351) | 53 (31%) |  |  | 0.843(0.599-1.187) |  |  | 0.697(0.465-1.043) |  |  | 0.827(0.546-1.251) |
|  | **T/T** | 14(0.046) | 19 (0.068) | 12 (7%) |  |  | 1.424(0.691-2.935) |  |  | 1.375(0.613-3.085) |  |  | 0.965(0.450-2.070) |
| **Dominant** | **C/C** | 170(0.556) | 162 (0.581) | 106 (62%) |  |  | Ref. |  |  | Ref. |  |  | Ref. |
|  | **T/T-T/C** | 136(0.444) | 117 (0.419) | 65 (38%) |  | 0.541 | 0.903(0.651-1.253) |  | 0.172 | 0.767(0.523-1.123) |  | 0.51 | 0.849(0.575-1.254) |
| **Recessive** | **T/C-C/C** | 292(0.954) | 260 (0.932) | 159 (93%) |  |  | Ref. |  |  | Ref. |  |  | Ref. |
|  | **T/T** | 14(0.046) | 19 (0.068) | 12 (7%) |  | 0.242 | 1.524(0.749-3.101) |  | 0.26 | 1.574(0.711-3.485) |  | 0.933 | 1.033(0.488-2.185) |

**Abbreviations:** Co, control; Freq., frequency; IT, initial-treatment; RT, retreatment; OR, odds ratio; CI, confidence interval.

Note: The *P*-value, OR and 95%CIs of pairs comparison between IT and control, RT and control, and RT and IT were calculated based on the logistic regression model adjusted by age and gender. Bonferroni correction was applied and the *P*-value was adjusted to 0.008 (0.05/6).

**sTable 3. CCR5 promoter haplotype frequencies comparisons between EPTB and PTB group**

| **Haplotype** ^a^ | | **Haplotype similarity** ^b^ | **EPTB patients (2n=250)** | **PTB patients (2n=650)** | ***P*** ^c^ | **OR (95%CI)** |
| --- | --- | --- | --- | --- | --- | --- |
|  |  |  | **N(freq.)** | **N(freq.)** |  |  |
| H1: | A A T G T C G C | HHC | 121.5(0.486) | 250.99(0.386) | 0.005 | 1.515(1.129-2.033) |
| H2: | A A G A C C A T | HHF*1 | 47.6(0.190) | 128.64(0.198) | 0.827 | 0.960(0.664-1.388) |
| H3: | G A G A C C A C | HHE | 45.9(0.184) | 95.95(0.148) | 0.181 | 1.301(0.884-1.915) |
| H4: | A A G G T C A C | HHA | 9.8(0.039) | 38.87(0.060) | 0.236 | 0.653(0.321-1.329) |
| H5: | A A G G T C G C | unknown | 11.2(0.045) | 49.95(0.077) | 0.078 | 0.552(0.283-1.079) |
| H6: | A A G G C C A T | unknown | 1(0.004) | 13.46(0.021) | 0.082 | 0.197(0.026-1.512) |
| H7: | A A G A T C G C | unknown | 3(0.012) | 12.88(0.020) | 0.416 | 0.595(0.168-2.106) |
| H8: | A A G A C C G T | unknown | 1.6(0.006) | 3.30(0.005) | 0.541 | 1.739(0.289-10.472) |
| H9: | A A T G C C G C | unknown | 0(0.000) | 1.01(0.002) | 0.833 | 1.295(0.117-14.343) |
| H10: | A A T A T C G C | unknown | 3(0.012) | 17.24(0.027) | 0.197 | 0.452(0.131-1.557) |
| H11: | G A G G C C A C | unknown | 0(0.000) | 10.51(0.016) | 0.102 | 0.212(0.027-1.643) |

**Note:** a: Haplotypes were constructed by rs2227010-rs2856758-rs2734648-rs1799987-rs1799988-rs41469351-rs1800023-rs1800024.

b: HHA, HHB, HHC, and HHF were previously reported (25-27).

c: Bonferroni correction was applied and the P-value was adjusted to 0.004 (0.05/11).


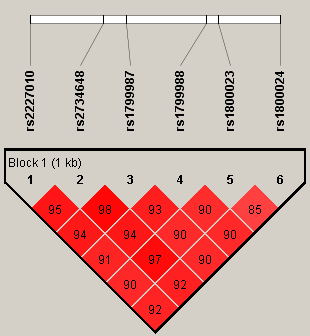


**sFigure 1 LD plot of SNPs in CCR5 promoter in Chinese Han population.**

*D’* value among SNPs were showed in matrix.
